# Extracellular DNA Containing (dG)n Motifs Penetrates into MCF7 Breast Cancer Cells, Induces the Adaptive Response, and Can Be Expressed

**DOI:** 10.1155/2019/7853492

**Published:** 2019-11-03

**Authors:** Ekaterina A. Kozhina, Elizaveta S. Ershova, Natalya A. Okorokova, Vladimir P. Veiko, Elena M. Malinovskaya, Vasilina A. Sergeeva, Marina S. Konkova, Serguey I. Kutsev, Nataly N. Veiko, Svetlana V. Kostyuk

**Affiliations:** ^1^Research Centre for Medical Genetics (RCMG), Moscow 115478, Russia; ^2^I.M. Sechenov First Moscow State Medical University (Sechenov University), Moscow, Russia; ^3^Bach Institute of Biochemistry, Biotechnology Research Center, Russian Academy of Sciences, Moscow 119071, Russia

## Abstract

**Background:**

Oxidized human DNA or plasmid DNAs containing human ribosomal genes can easily penetrate into the breast cancer cells MCF7 and stimulate the adaptive response induction. Plasmid DNA containing a CMV promoter, gene *EGFP*, and the insertion of the human ribosomal genes can be expressed. A hypothesis is proposed: these features of the ribosomal DNA are due to the presence of dGn motifs that are prone to oxidize.

**Methods:**

Cells of MCF7 line were cultured with plasmids which contained a CMV promoter and gene of fluorescent protein EGFP. Genetic construction pEGFP-Gn contains pEGFP vector and a small insertion with dG_11_ and dG_13_ motifs that are inclined to oxidation. The accumulation of pEGFP and pEGFP-Gn in MCF7 (qPCR), the levels of ROS in the cells, the content of 8-oxodG in plasmids and cellular DNA (flow cytometry, immunoassay, and fluorescent microscopy), the expression of *NOX4* and *EGFP*, the localization of NOX4 and EGFP in MCF7 (qPCR, flow cytometry, and fluorescent microscopy), and the levels of the cell DNA damage (comet assay) were analyzed.

**Results:**

(dG)n insertions in the plasmid pEGFP increase the levels of ROS, the cell DNA oxidation and DNA damage, and the level of transfection of plasmid into the MCF7 cells. NOX4 participates in the oxidation of pEGFP-Gn and pEGFP. The expression of *EGFP* gene in MCF7 is significantly increased in case of pEGFP-Gn. Stimulation of ROS synthesis (H_2_O_2_ 40 *μ*M or 10 cGy IR) increases the level of expression of *EGFP*.

**Conclusions:**

GC-rich DNA fragments containing dGn motifs that are inclined to oxidation penetrate into MCF7 cancer cells, stimulate the adaptive response, and can be expressed. This property of GC-rich cell-free DNA should be considered and/or could potentially be used in therapy of tumors.

## 1. Background

Cell-free DNA (cfDNA) circulating in the bloodstream is widely used for cancer diagnosis [1–3]. In recent years, there is increasing evidence of the biological activity of circulating cfDNA fragments with respect to different types of human cells [4]. Nucleic acids, which are released by damaged and dying cells, are also recognized by some sensors of different nature, termed pattern recognition receptors (PRRs), and belong to the damage-associated molecular pattern (DAMP) family [5, 6]. These DAMPs enter into the composition of circulating cfDNA and underlie the biological activity of cfDNA. For example, it was shown that circulating cfDNA promotes colorectal tumor cell survival after cytotoxic chemotherapy [7].

One of the features of circulating cfDNA is the increased level of oxidation [8, 9]. The *in vitro* experiments have shown that an exposure to the human oxidized DNA enhances both instability of genome and survival in MCF breast cancer cells [9, 10]. The effect of oxidized cell-free DNA (oxo-cfDNA) can be explained by the ability to easily penetrate into the cancer cells. oxo-cfDNA induces synthesis of ROS in the mitochondria. This leads to oxidation of nuclear DNA, and DNA breaks and causes DNA damage response (DDR) and adaptive response [10]. We recently showed that plasmid DNA fragments containing human ribosomal genes could also penetrate into the MCF7 and be expressed [11].

**Figure 1:**
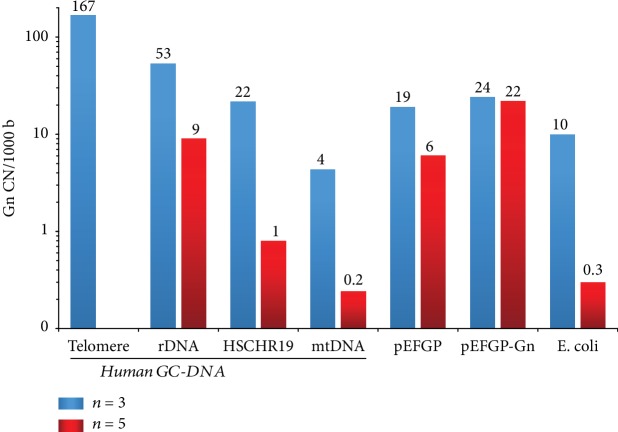
The content of dGn motifs in the GC-DNAs. The DNAs analyzed are indicated in the graph. The fragment DNA designated as HSCHR19: a randomly chosen GC-rich fragment of Homo sapiens chromosome 19 genomic scaffold, GRCh38 (NCBI Reference Sequence: NT_011295.12).

Analysis of human ribosomal repeat sequence revealed that the transcribed region of human ribosomal repeat (TR-rDNA) contains many dGn motifs (Figure 1). In general, GC-rich regions of human nuclear DNA differ from human mtDNA or GC-rich bacterial DNA by the presence of a large number of dGn motifs. The nucleoside dG inside dGn has the lowest oxidation potential among all nucleosides in DNA [12]. Circulating cfDNA fragments containing these motifs should be easily oxidized and exhibit activity that is a characteristic of oxidized DNA.

So, we can assume that, regardless of the sequence, any DNA fragments containing (dG)n motifs will stimulate ROS generation, penetrate into the cells, induce the adaptive response, and will be expressed. We confirmed this hypothesis by examining a bacterial plasmid that contained (dG)_11_ and (dG)_13_ inserts.

## 2. Methods

### 2.1. Cell Culture

#### 2.1.1. Cancer Cells

ER/PR-positive MCF7 cells are purchased at ATCC, Manassas, USA (Cat: HTB-22). Human astrocytoma cells (1321N1) were obtained from the RCMG collection. Cells are cultured in DMEM with 10% (*v*/*v*) fetal calf serum, 2 mM L-glutamine. The medium contains 100 *μ*g/mL of streptomycin and 100 units/mL of penicillin. MCF7 cells were grown in humidified atmosphere at 37°C. The atmosphere contained CO_2_ (5%). Before treatment with plasmids, cells were grown for 24 h in slide flasks.

#### 2.1.2. Noncancerous Cells

Human embryonic lung fibroblasts (HEFs) and human mesenchymal stem cells (MSCs) were obtained from the RCMG collection. HEFs were cultured in Eagle's medium with 10% fetal bovine serum. MSCs were cultured in F10 (Invitrogen) complemented with 20% fetal bovine serum (FBS), 2 mM glutamine, 10 mM HEPES, 100 U/mL penicillin, 100 mg/mL streptomycin, 10-6 M dexamethasone, and 2.5 ng/mL basic fibroblast growth factor (FGF) (Sigma-Aldrich).

Ethical approval for the use of primary human cells was obtained from the Committee for Medical and Health Research Ethics of the Research Centre for Medical Genetics (approval number 5).

### 2.2. Irradiation of Cells

The cells were exposed to X-ray irradiation (10 cGy) at the unit ARINA-2 (Spectroflash, St. Petersburg, Russia). The amplitude of voltage at the X-ray tube was ∼160 kV and was at a maximum in the center of radiation ∼60 keV. The dosage rate amounted to 0.16 Gy/min.

### 2.3. Plasmid pEGFP and pEGFP-Gn (GC-DNAs)

**Figure 2:**
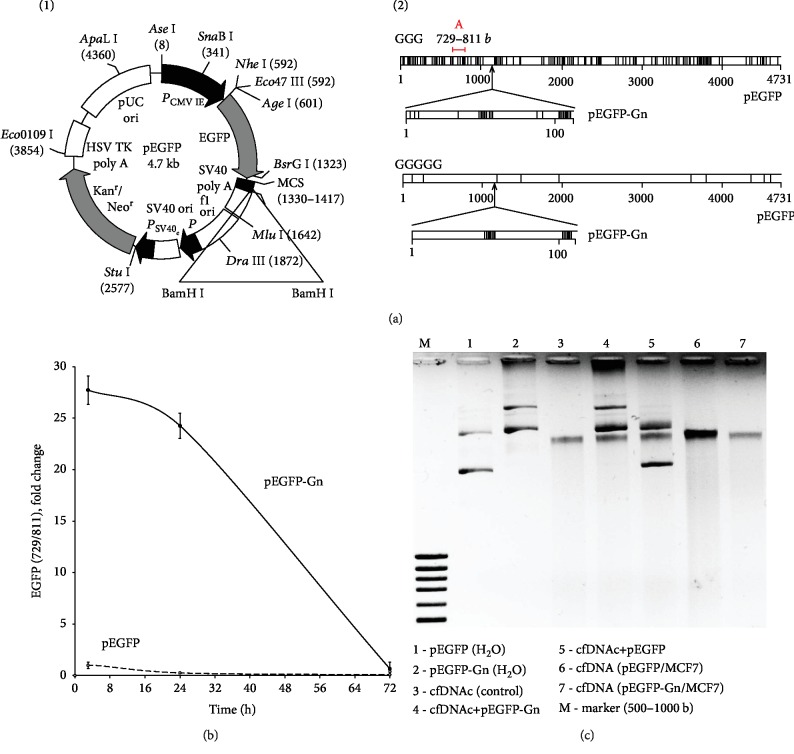
Some characteristics of the plasmids pEGFP and pEGFP-Gn. (a) Plasmid pEGFP-C1 (pEGFP) (http://www.bdbiosciences.com). (2) Distribution of dG_3_ and dG_5_ motifs within the pEGFP and pEGFP-Gn. Fragment A (red line), used for the analysis of plasmid DNA and RNA *EGFP*, is indicated. (b) Quantitation of pEGFP and pEGFP-Gn in the cellular DNA after 3, 24, and 72 hours of incubation; pEGFP content (3 h) is taken as one unit. (c) Plasmid content and fragmentation in the extracellular DNA after 3 hours of MCF7 incubation with 100 ng/mL pEGFP or pEGFP-Gn. Electrophoresis of DNA was carried out in a 1% agarose gel stained with ethidium bromide. Gel tracks 4 and 5—plasmids were added to the medium immediately prior to isolation of the cfDNA.

Plasmid pEGFP-C1 (pEGFP, 53.4% GC) that contains the gene of EGFP (http://www.bdbiosciences.com, GenBank accession number U55763) was used as a vector (Figure 2(a)). Inserted DNA fragment was synthesized and consisted of 110 base pairs flanked with BamHI restriction sites and containing the dG_13_ and dG_11_: 5′gatccaccggatctagataatcgccgtcccgcccgccgcctt**ggggggggggggg**, 3′gtggcctagatctattagcggcagggcgggcggcggaaccccccccccccc, atacaccggatctagataatcgccgtcccgcccgccgcctt**gggggggggg** 3′, tatgtggcctagatctattagcggcagggcgggcggcggaaccccccccccctag 5′, BamHI.

The vector pEGFP-C1 was treated with BamHI and added to the DNA fragment with subsequent ligation with T4 DNA ligase. Competent E. coli (strain JM110) were then transformed and grown on LB with agarose and kanamycin (50 *μ*g/mL). The clones were analyzed by PCR. Selected clones were grown in liquid medium and plasmids were isolated. After confirmation of the designed DNA sequence by sequencing, the plasmids were extracted using an Invisorb Plasmid Maxi Kit (http://www.invitek.de).

All the GC-DNA samples were purified with the EndoFree Plasmid Purification kit.

(http://www.qiagen.com/). In order to show that the observed response was caused exclusively by DNA, not by endotoxin residuals, we provided additional experiments. (1) A sample of plasmid DNA underwent complete hydrolysis down to nucleosides. For this, we used DNA exonucleases and phosphatase. These plasmid hydrolysates did not show such biological activity as intact DNA. (2) We analyzed the expression of TLR4 gene, which, as we know, is always activated in the presence of the endotoxin. The plasmid DNA did not induce an increase of TLR4 expression.

### 2.4. Flow Cytometry

Cells were washed in Versene solution and treated with 0.25% trypsin under light microscopic observation. Cells were transferred to the Eppendorf tubes, washed with the culture media, then centrifuged, and suspended in PBS.

#### 2.4.1. EGFP

We analyzed nonfixed cells at 488 nm (blue laser). To quantify the background fluorescence, the control cells were analyzed.

#### 2.4.2. 8-oxodG, NOX4

Staining of the cells with various antibodies was performed as described below. Cells were fixed with 2% paraformaldehyde (Sigma) at 37°C for 10 min, washed three times with 0.5% BSA-PBS, and permeabilized with 0.1% Triton X-100 (Sigma) in PBS for 15 min or with 70% ethanol at 4°C. Cells (~50 × 10^3^) were washed three times with 0.5% BSA-PBS and stained with 1 ***μ***g/mL PE-8-oxodG or FITC-8-oxodG antibody, PE-NOX4, PE-AIM2 antibodies (Abcam) for 3 h at 4°C, then again washed thrice with 0.5% BSA-PBS. We stained a portion of the cells with only secondary FITC- (PE-) conjugated antibodies to estimate the background fluorescence. We analyzed the cells with CyFlow Space (Partec, Germany).

### 2.5. Quantification of mRNA Levels

We isolate total mRNA using RNeasy Mini kits (Qiagen, Germany). It was treated with DNAse I and reverse transcribed with Reverse Transcriptase kit (Sileks, Russia). The expression profiles were obtained with qRT-PCR with SYBR Green PCR Master Mix (Applied Biosystems). We used the StepOnePlus (Applied Biosystems) to analyze mRNA levels. The technical error is approximately 2%. The following primers were used (Sintol, Russia): *NOX4* (F: TTGGGGCTAGGATTGTGTCTA; R: GAGTGTTCGGCACATGGGTA), *EGFP* (F: TACGGCAAGCTGACCCTGAAG; R: TGAAGCACTGCACGCCGTAGG), and *TBP* (as a reference gene) (F: GCCCGAAACGCCGAATAT; R: CCGTGGTTCGTGGCTCTCT).

According to our data, in the MCF7-plasmid system, the TBP and B2M genes are suitable as controls. The expression of these genes is almost unchanged under the conditions used.

### 2.6. Quantification of pEGFP and pEGFP-Gn in the Cells and Medium

#### 2.6.1. The Cells

After medium removal by centrifugation at 460 g, we washed the cells in Versene solution, then treated with trypsin (0.25%), and transferred into Eppendorf tubes. Cells were suspended in the solution (1 mL) containing sodium lauryl sarcosylate (0.2%), EDTA (0.002 M), and 75 *μ*g/mL RNAse A (Sigma, USA). It was incubated during 45 min and treated with proteinase K (200 *μ*g/mL, Promega, USA) for 24 h at 37°C. After two cycles of the purification using saturated phenolic solution, we precipitate DNA fragments by adding two volumes of ethanol in the presence of ammonium acetate (2 M). The precipitate was then washed with 75% ethanol twice. Then, it dried and dissolved in water. To determine DNA concentration, we measured fluorescence intensity with PicoGreen (Molecular Probes/Invitrogen, CA, USA). pEGFP and pEGFP-Gn were obtained using qPCR with SYBR Green PCR Master Mix (Applied Biosystems). We used the following primers (Sintol, Russia): *EGFP* (F: TACGGCAAGCTGACCCTGAAG; R: TGAAGCACTGCACGCCGTAGG); *Human B2M* (as a reference gene, accession number M17987): F: GCTGGGTAGCTCTAAACAATGTATTCA; R: CCATGTACTAACAAATGTCTAAAATGG.

#### 2.6.2. Incubation Medium

To extract DNA from the cell culture medium, we used a procedure similar to the described above for the cells. Electrophoresis of DNA was conducted in a 2% agarose gel. The gel was stained with ethidium bromide.

### 2.7. 8-oxodG Levels in pEGFP and pEGFP-Gn

**Figure 3:**
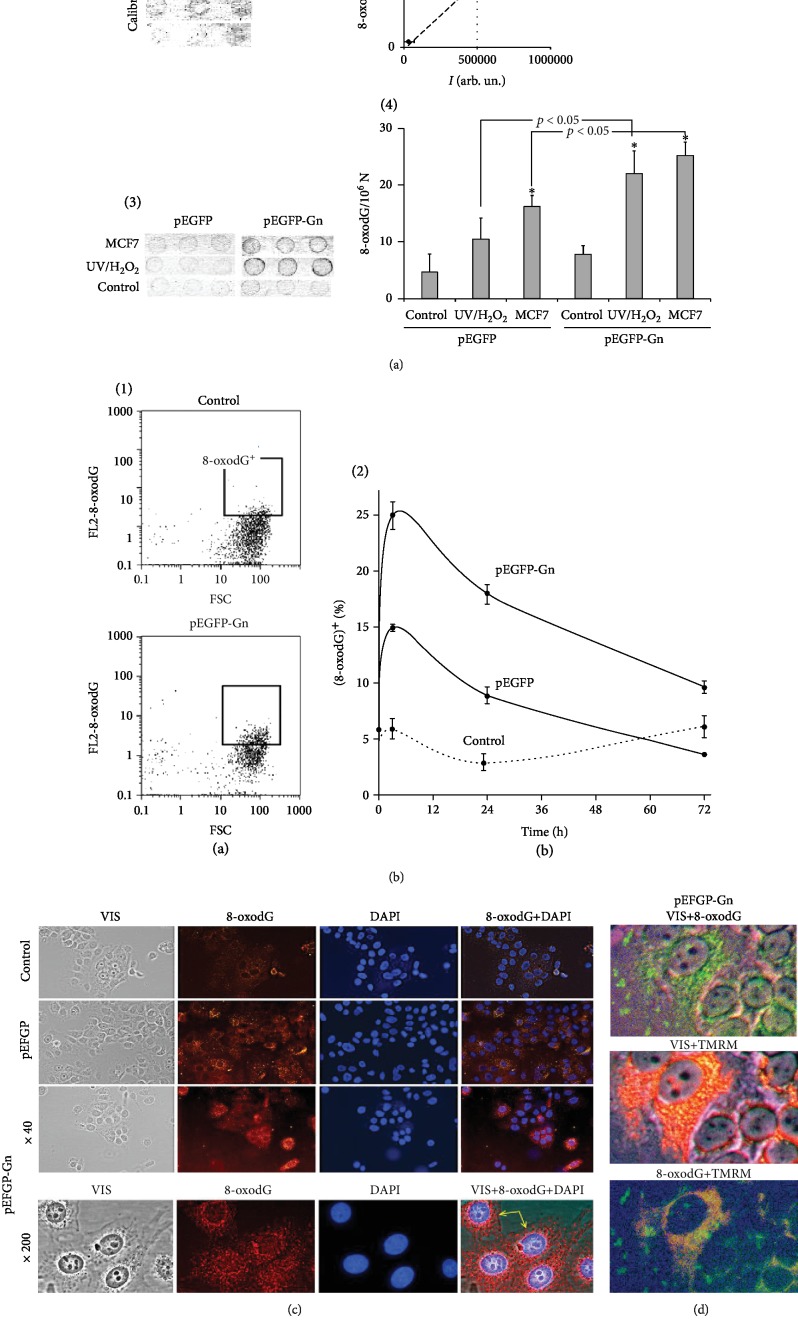
8-oxodG levels in MCF7 and GC-DNAs. (a) 8-oxodG levels in pEGFP and pEGFP-Gn. The method of immunoassay using 8-oxodG antibodies conjugated with alkaline phosphatase was used. (1, 2) Standard samples of the oxidized genomic DNA (10 ng/dot) with a known content of 8-oxodG were applied in order to plot a calibration curve for the dependence of the signal intensity on the number of 8-oxodG copies. (3, 4) The samples of the oxidized and nonoxidized (control) pEGFP and pEGFP-Gn (10 ng/dot) were applied. MCF7: GC-DNAs after 3 hours of incubation with MCF7; UV/H_2_O_2_: plasmids were oxidized in 0.1% H_2_O_2_ solution with UV irradiation (*λ* > 312 nm). (b) FCA. (1) Cell plots: FL2 (8-oxodG-PE) versus FCS. (8-oxodG)^+^: gated area. Graph: relative proportions of 8-oxodG positive cells (change with time). (c) FM. Evaluation of 8-oxodG (PE, red) in the cells treated with GC-DNAs (3 h). Yellow arrows indicate the surface of the cells, where it is possible to localize the granules of oxidized DNA. (d) FM. 8-oxodG (FITC, green) and mitochondria (mitotracker TRMR, red) in the cells treated with pEGFP-Gn (1.5 h). The mitochondria were analyzed in nonfixed cells with TRMR. The cells were then fixed and analyzed for 8-oxodG. Magnification ×200.

MCF7 3 h (Figure 3(a)). MCF7 cells were cultured with plasmids in the medium for three hours. The RNA fraction was isolated with YellowSolve (Sileks, Russia). The RNA fraction contained fragments of plasmid DNA. RNA was digested (1 h, 37°, 75 *μ*g/mL RNAse A); DNA was precipitated with 75% ethanol.

UV/H_2_O_2_ (Figure 3(a)). The method of DNA oxidation was described previously in [5]. Plasmids pEGFP and pEGFP-Gn (100 ng/*μ*L) were oxidized in 0.1% H_2_O_2_ solution using UV irradiation (*λ* > 312 nm) for 3 minutes at 25°C. This DNA was precipitated with 2 volumes of ethanol at the presence of 2 M ammonium acetate. The precipitate was washed for two times with 75% ethanol, dried, and dissolved with water. Resulting DNA concentrations were assessed by UV spectra.

Quantitation of 8-oxodG was described in [3]. DNA samples were applied to a filter (Optitran BA-S85, GE healthcare). Three dots (10 ng/dot) were applied for each sample. Four oxidized genomic DNA samples (10 ng/dot) with a known level of 8-oxodG (was determined with ESI-MS/MS using AB SCIEX 3200 Qtrap machine [3]) were applied to the same filter to plot a calibration curve for the dependence of signal intensity on the quantity of 8-oxodG copies in a particular sample. The filter was heated in vacuum during 1.5 h at 80°С. 8-oxodG antibody conjugated with alkaline phosphatase was used. After that, the filter was placed into a solution of substrates for alkaline phosphatase NBT and BCIP. After the completion of reaction, we washed the filter with water and it was dried in the darkness. Then, the filter was scanned. We used special software (Images6, RCMG, Moscow) for quantitative analysis of the dots. Signals from several dots for the same sample were averaged. The 8-oxodG level in a sample was estimated using the calibration curve equation. Relative standard error was 15 ± 5%.

### 2.8. DNA Damage Evaluation Using Comet Assay

The degree of the DNA damage was analyzed by means of the DNA-comet assay using alkaline conditions. As the basis, it was used a special protocol of the aforementioned micromethod developed by the German company “Trevigen, Inc.,” including minor modifications. In order to prepare the slides, the cell suspension in a 1% solution of the low-melting-point agarose was applied onto a “base” made of the normal-melting-point agarose (NGA). The electrophoresis was performed at the current of 9-12 mA (0.7-1.5 V/сm) for 20 min, followed by fixing the slides in a 96% ethanol. In order to visualize the DNA comets, the slides were stained with PI (Sigma) (2 *μ*g/mL in PBS). The quantitative parameters were measured using an Axiophot microscope (Zeiss) and DCM 300 digital video camera (Russia). 200-400 nuclei per each slide were analyzed. The software package СometScore v. 1.5 (supplied by TriTek Corp., http://tritekcorp.com) facilitated the comet assay performance. In order to analyze the DNA comets, such quantitative characteristic as the comet tail moment was used (resulting from multiplying the comet's tail length by the DNA percentage in the tail).

### 2.9. ROS Assay

We analyzed the cells using total fluorescence assay in the 96-well plate format at *λ*_ex_ = 488 nm and *λ*_em_ = 528 nm (EnSpire equipment, Finland). The medium was replaced by 5 *μ*m H2DCFH-DA (Molecular Probes/Invitrogen, CA, USA) in PBS solution, and a relative fluorescence intensity increase was detected at 37°C. Eight (4 × 2) repeated measurements were done for each GC-DNA and 16 for the control. The mean absolute intensities of fluorescence were divided by the average value of the intensity corresponding to *t* = 0, obtaining the values of *I*_0_. To present the graphs, we used coordinates *I* − time.

### 2.10. Statistics

All reported results for PT-qPCR, qPCR, FCA, and immunoassay were reproduced at a minimum of three times as independent biological replicates. To analyze the significance of the observed differences, we used the nonparametric Mann-Whitney *U*-tests. The data were analyzed using StatPlus2007 Professional software (http://www.analystsoft.com/). All *p* values are considered statistically significant at *p* < 0.05. The software for “Imager 6” was designed by Roman Veiko (RCMG, Moscow).

## 3. Results

### 3.1. Constructions

A genetic construction pEGFP-Gn was synthesized to study the functional role of dGn motifs. It contains the vector pEGFP and a small insertion with two dGn ((dG)_11_ and (dG)_13_) motifs (Figure 2(a), 2). These motifs are prone to selective oxidation [12]. BamHI restriction site is included in the multiple cloning sites (MCS, Figure 2(a)) that encode the C-terminal fragment of the EGFP protein. This approach allows to clone fragment in the vector and to avoid the disruption of the structure of functionally important sites. Figure 2(a), 2 shows the distribution of GGG and GGGGG in both plasmids. Only pEGFP-Gn contained longer (dG)n sequences.

### 3.2. The Amount of pEGFP and pEGFP-Gn within the Cells

Quantitative analysis of the DNA region common for both plasmids was performed with qPCR (fragment A: 729–811 nucleotide, gene *EGFP*, Figure 2(a)). This region has no homology with human DNA. Cells were cultured for 24 hours, then plasmids were added to the medium (100 ng/mL). Cells were incubated for 3, 24, or 72 hours (Figure 2(b)). Total cell DNA was then isolated. The maximum amount of plasmids in the cells was determined after 3 hours of incubation. The amount of the fragment A was approximately 30 molecules per cell for the pEGFP and 700 molecules per cell for the pEGFP-Gn. After 72 hours of incubation, the qPCR signal was not significantly different from background values. Thus, insertion of oligo(dG) into the genetic construction causes an approximately 20-fold increase in the binding of pEGFP-Gn plasmid to the cells compared to the vector itself.

Analysis of the cfDNA from the culture medium supports this conclusion (Figure 2(c)). The amount of cfDNA in the medium of control MCF7 was 110 ± 20 ng/mL (track 3). The amount of cfDNA after 3 hours of culturing cells in the presence of pEGFP was 190 ± 30 ng/mL (track 6) and in the presence of pEGFP-Gn was 100 ± 20 ng/mL (track 7). It is important to note that after 3 hours, we did not observe supercoiled and circular forms of plasmids in the medium, which are usually present in aqueous solution (track 1, 2 and 4, 5). Plasmid pEGFP is highly fragmented (track 6); plasmid pEGFP-Gn is almost not visible in the gel (track 7). Thus, we can conclude that (1) plasmids degrade rapidly in the culture medium and (2) pEGFP-Gn is almost completely associated with the cells.

### 3.3. GC-DNAs Induced ROS Synthesis in MCF7 Cells

**Figure 4:**
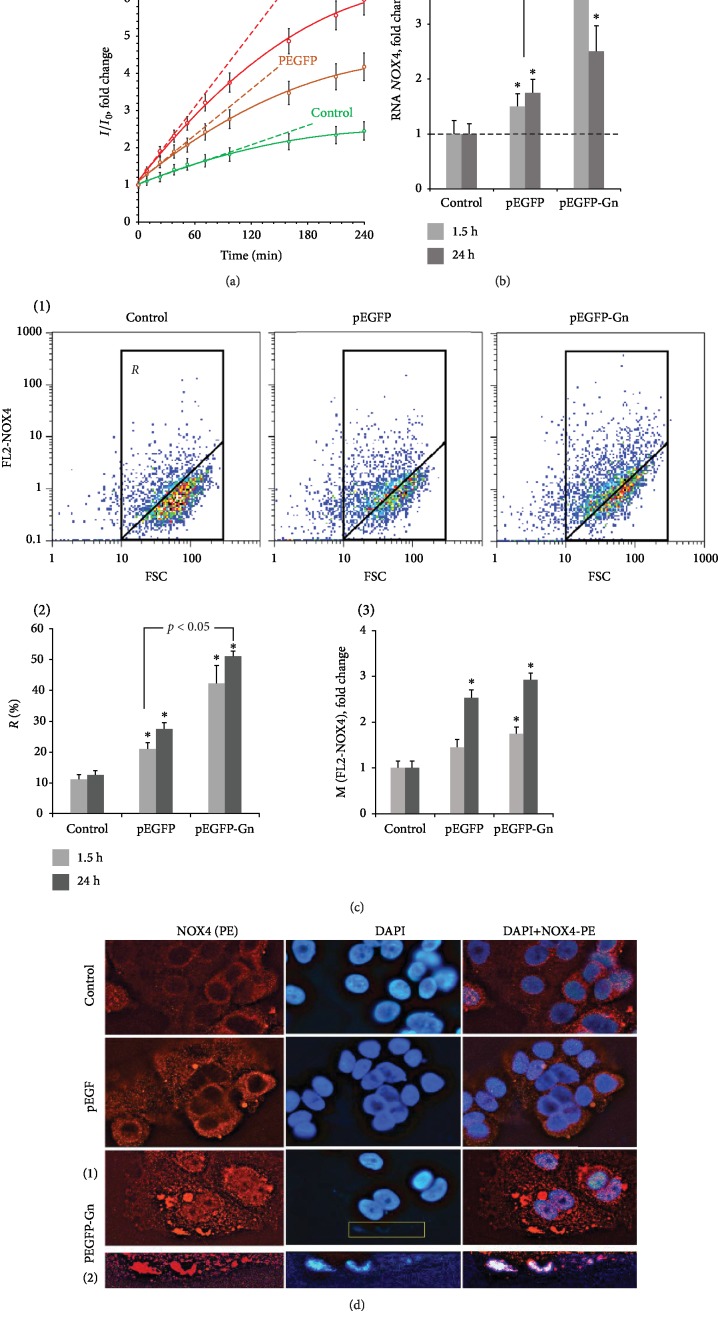
The exposure of MCF7 cells to pEGFP and pEGFP-Gn leads to an increase in ROS production. (a) FL-plate reader. The cells were analyzed in the 96-well plate format at *λ*_ex_ = 488 nm and *λ*_em_ = 528 nm. The cultivation medium was replaced with 5 *μ*m H2DCFH-DA in PBS solution, 50 ng/mL GC-DNA was immediately added, and a fluorescence intensity increase was detected at 37°C. *I*, *I*_0_—sample's signal at time *t* and immediately after GC-DNA addition, respectively. (b) RT-qPCR. The levels of RNA NOX4 in the cells exposed to pEGFP or pEGFP-Gn for 1.5 h and 24 h. The data are normalized to the content of RNA NOX4 in the control. (c) FCA. (1) Cell plots: FL2 (NOX4-PE) versus FSC. *R*: gated area. (2) Proportions of NOX4+ cells in *R* gate. (3) Median signal intensity of FL2-NOX4. (d) FM-based evaluation of NOX4 (PE) in the cells treated with pEGFP-Gn or pEGFP (1.5 h). Magnification ×60. pEGFP-Gn (2): for DNA analysis of the selected area of the photo (1), the image size and brightness were increased by computer processing. ^∗^*p* < 0.05.

The primary MCF7 cell response to a change of the DNA content in the culture medium is ROS synthesis, Figure 4(a). The ROS synthesis was analyzed using the reaction of DCF formation [13]. The kinetics of DCF formation upon the exposure of the cells to GC-DNAs was examined using a fluorescent reader. The pEGFP-Gn induced ROS synthesis to a greater extent than pEGFP. The rate of ROS synthesis began to grow immediately after adding DNA samples to the medium. As quickly as 30 minutes later, the rate of ROS synthesis considerably reduced, Figure 4(a).

The major source of ROS in MCF7 is NAD(P)H oxidases, predominantly one encoded by the *NOX4* [14]. Using qRT-PCR, we profiled the RNA levels of *NOX4* in the control cells and the cells exposed to pEGFP-Gn or pEGFP (Figure 4(b)). After 1.5 hours of MCF7 culturing with pEGFP-Gn or pEGFP, the amount of RNA *NOX4* increases 3.7 and 1.8 times, respectively.

The amount of NOX4 protein was assessed with FCA. Two parameters were analyzed: the content of NOX4^+^ cells (gate *R*, Figure 4(c), 1 and 2) and the median values of signal (Figure 4(c), 3). After 1.5 hours, the number of NOX4^+^cells increases from 10% (control) to 42% (pEGFP-Gn) and 21% (pEGFP). After 24 h of incubation, the number of NOX4^+^ cells remains practically unchanged (Figure 4(c), 2), but the average signal level increases (Figure 4(c), 3); thus, more cells are expressing NOX4 in elevated quantities compared to control.

FM confirmed these data (Figure 4(d)). The fluorescence intensity of the cells stained with NOX4(PE) antibody increases in the presence of the plasmids. NOX4 is localized in the cytoplasm. NOX4 is also detected in the area of the nuclei in the presence of pEGFP-Gn. In addition, pEGFP-Gn stimulates the formation of platform-like structures of NOX4 in the close proximity to the cell membrane. Staining with DAPI (a dye that stains DNA) shows that some cells have weak blue signals, indicating the conglomerates of DNA molecules on the cell surface (Figure 4(c), pEGFP-Gn (2)). A large number of NOX4 molecules are localized in the area of these blocks of DNA. Thus, pEGFP-Gn causes an increase in the expression of NOX4 on the membrane, in the cytoplasm, and in the nuclei of cells. pEGFP stimulates the expression of NOX4 to a lesser degree.

### 3.4. Oxidation of pEGFP and pEGFP-Gn

To analyze the content of 8-oxodG in the GC-DNAs, the method of immunoassay was used (Figure 4(a)). pEGFP and pEGFP-Gn were oxidized in a solution of 0.1% H_2_O_2_ under UV irradiation (*λ* > 312 nm) or added for 3 hours to the culture medium of MCF7. Plasmids were isolated from the cells in the fraction of RNA (see Methods). Hydrolysis with RNase I was then performed and the DNA analyzed (Figure 3(a), 3). In both experiments, pEGFP-Gn contained more 8-oxodG than pEGFP (Figure 3(a), 3 and 4). After incubation for 3 hours with the cells of 8-oxodG, pEGFP-Gn and pEGFP contained 0.12 and 0.07 of 8-oxodG per plasmid molecule, respectively.

### 3.5. The Content of 8-oxodG in MCF7

The content of 8-oxodG in MCF7 was measured with flow cytometry (FCA) using antibodies to 8-oxodG, conjugated with PE (Figure 3(b)). The content of the cells with a high level of 8-oxodG ((8-oxodG^+^), Figure 3(b), 1) was analyzed. In three hours, the proportion of cells 8-oxodG^+^ in the presence of pEGFP-Gn or pEGFP reaches maximum (25% and 15%). The number of the cells of this fraction is reduced after 24 and 72 hours.

Figure 3(c) shows the localization of 8-oxodG in the cells after 24 hours of incubation with pEGFP-Gn and pEGFP. The fluorescent signals are localized predominantly in the cytoplasm (mitochondria). Weak staining of the nuclei and the areas of the cell membrane can be observed in the presence of pEGFP-Gn (yellow arrows on Figure 3(c) (×200)).

Figure 3(d) shows the localization of 8-oxodG and mitochondria in the cells 1.5 hours after the incubation with pEGFP-Gn. Most of the signals of 8-oxodG (antibodies for 8-oxodG conjugated with FITC) are located in the cytoplasm and are colocalized with the mitochondria (mitotracker TMRM). About 20% of the cells have a weak fluorescent signal near the cell membrane, which seem to reflect the location of the oxidized pEGFP-Gn (green spots, Figure 3(d)).

### 3.6. DNA Damage

**Figure 5:**
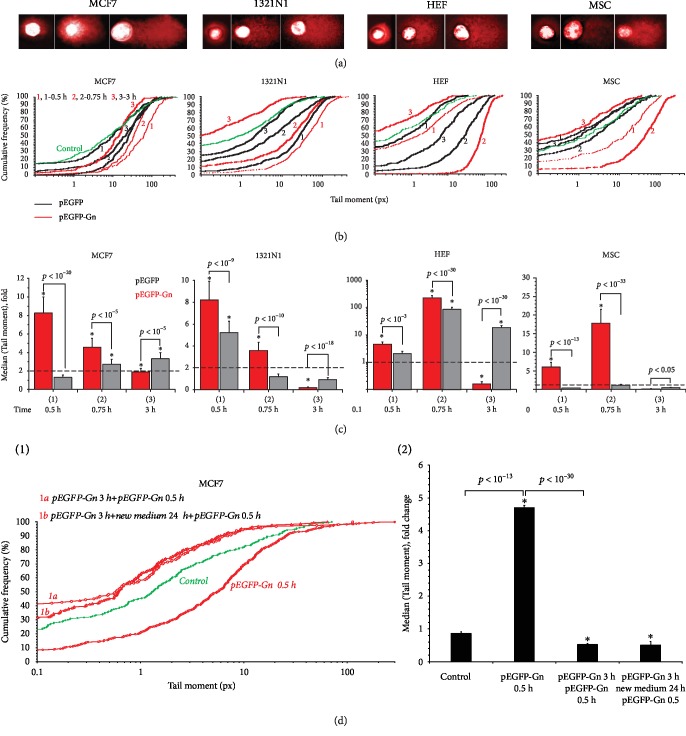
An exposure of the human cultured cells to GC-DNAs leads to an increase in DNA damage. (a) Comet assay in alkaline conditions. Digital photography of the nuclei with varying degree of DNA damage. (b) Cumulative histograms for the tail moment (TM) of the comets. The time of incubation of the cells with GC-DNAs and the type of GC-DNA are shown on the figure. (c) The average of the median (TM) for the comet assay. (d) Cumulative histograms for the tail moment (TM) of the comets (1) and the average of the TM (2). 1a: the cells were incubated for 3 hours in the presence of 50 ng/mL pEGFP-Gn, then pEGFP-Gn was added for 0.5 hours. 1b: the cells were incubated for 3 hours in the presence of 50 ng/mL pEGFP-Gn, then the culture medium was changed, and after 24 hours, the pEGFP-Gn was added for 0.5 hours. ^∗^*p* < 0.05.

The level of nuclear DNA breaks was evaluated using comet assay (alkaline electrophoresis), Figure 5(a). In 30 min after adding pEGFP-Gn to the cell medium, DNA breaks were detected in the most cells (“comets” were formed, Figures 5(b) and 5(c)), but 3 hours later, the number of breaks considerably reduced. pEGFP also stimulated break formation, but the effect was much weaker (Figure 5).

The transient formation of DNA breaks under the short-term action of GC-DNAs on the MCF7 cells seemed to us an intriguing fact. Therefore, we tested the response of other cell cultures (cancerous and noncancerous) to the presence of the GC-DNAs in the culture medium. Human astrocytoma cells (1321N1) responded to the GC-DNAs as well as MCF7 cells. The maximum level of DNA breaks was detected after 30 minutes of cell incubation with 50 ng/mL pEGFP-Gn.

Human embryonic lung fibroblasts (HEFs) and human mesenchymal stem cells (MSCs) responded to the action of 50 ng/mL GC-DNAs by forming a large number of breaks 45 minutes after the start of incubation. In the case of noncancerous cells, the response to the action of pEGFP-Gn developed more slowly but was stronger than for the cancer cells. After 3 hours of 1321N1, HEF, and MSC incubation in the presence of pEGFP-Gn, the number of breaks decreased much lower than the control level.

To understand how long the cells “remember” the single action of pEGFP-Gn, we conducted the following two experiments (Figure 5(d)). First, we cultured the cells within three hours with 50 ng/mL pEGFP-Gn and then added 50 ng/mL pEGFP-Gn again for 30 minutes (curve 1a, Figure 5(d)). Secondly, we cultured the cells for three hours in the presence of 50 ng/mL pEGFP-Gn and then changed the cell culture medium to fresh. The cells were cultured for 24 hours, and pEGFP-Gn (50 ng/mL) was again added to the culture medium for 30 minutes (curve 1b, Figure 5(d)). For comparison, cells were only cultured within 30 minutes in the presence of 50 ng/mL pEGFP-Gn (curve 1, Figure 5(d)). We found that the number of breaks increased significantly only with a single action of pEGFP-Gn within 30 minutes. Repeated addition of pEGFP-Gn after 3 hours or 24 hours did not lead to an increase in the level of the DNA breaks. In contrast, the level of DNA damage was significantly reduced compared with the control.

### 3.7. An Exposure to GC-DNAs Enhances Survival in MCF7 Cancer Cells

**Figure 6:**
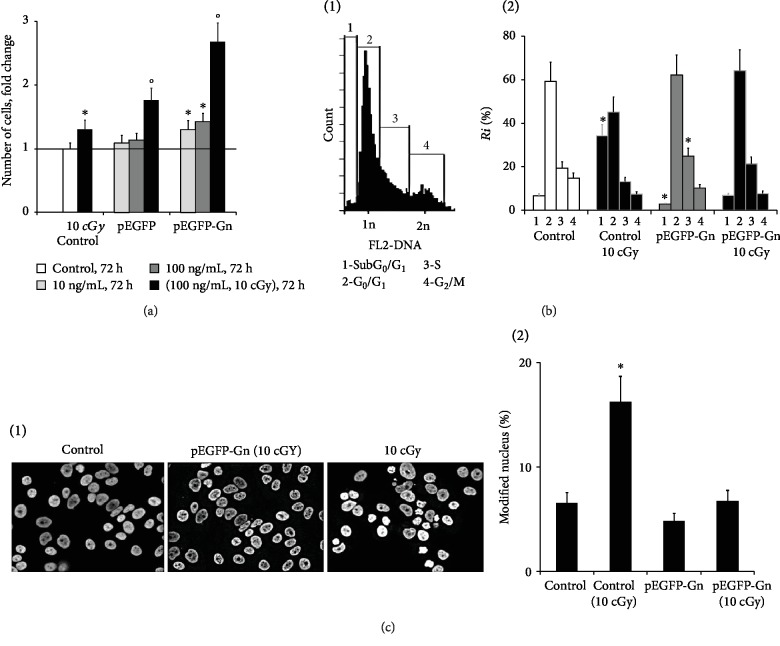
An exposure to pEGFP-Gn enhances survival in MCF7 cancer cells. (a) Total number of cells in studied cell population. Gray bars: plasmids were added to the cell culture medium and the cells were cultured for 72 hours. Black bars: plasmids were added to the cell culture medium; the cells were incubated for 30 min, then irradiated (10 cGy). After this, the cells were cultured for 72 hours. (b) FCA. (1) Distribution of fluorescence intensities of the cells stained with PI. (2) Proportions of the cells with the amount of DNA corresponding to the sub-G_0_/G_1_, G_1_-, S, and G_2_/M phases of the cell cycle. (c) Evaluation of modified nuclei in MCF-7. (1) Example of Hoechst 33342 staining; (2) graph of the proportion of cells with modified nuclei in four studied types of MCF-7 cultures. ^∗^*p* < 0.05.

After 72 hours of MCF7 cultivation in the presence of 10 or 100 ng/mL of pEGFP-Gn or pEGFP, the amount of the cells increases 40 and 15%, respectively (Figure 6(a)). When cells incubated with GC-DNAs for 1 hour are subjected to low-dose ionizing radiation (10 cGy), this response is even more prominent. Low dose radiation stimulates a 30% increase in the amount of cells in the control. In the samples treated with 100 ng/mL pEGFP-Gn/10 cGy or 100 ng/mL pEGFP/10 cGy, the amount of the cells increased to 1.8- and 2.5-fold, respectively (Figure 6(a)).

The effect of exposure to pEGFP-Gn and pEGFP-Gn/10 cGy on the cell cycle was studied on MCF-7 cells that were harvested 24 hours after addition of pEGFP-Gn (100 ng/mL) to the medium (Figure 6(b)). The cell count was performed after DNA-specific propidium iodide (PI) staining. In the population of the irradiated cells, the amount of the cells in the sub-G_0_/G_1_ phase was increased. pEGFP-Gn significantly decreased the amount of cells in this phase and increased the amount of cells in S-phase.

Nuclear morphology was evaluated after staining the cells with Hoechst 33342 (Figure 6(c)). The cell was marked as damaged if condensed nucleus with micronuclei and fragmented chromatin was detected. In the control, the amount of damaged cells increases after irradiation. In cells exposed to pEGFP-Gn/10 cGy (24 hours, 100 ng/mL), the amount of damaged cells decreased threefolds (Figure 6(c), 2).

### 3.8. EGFP Expression in MCF7

**Figure 7:**
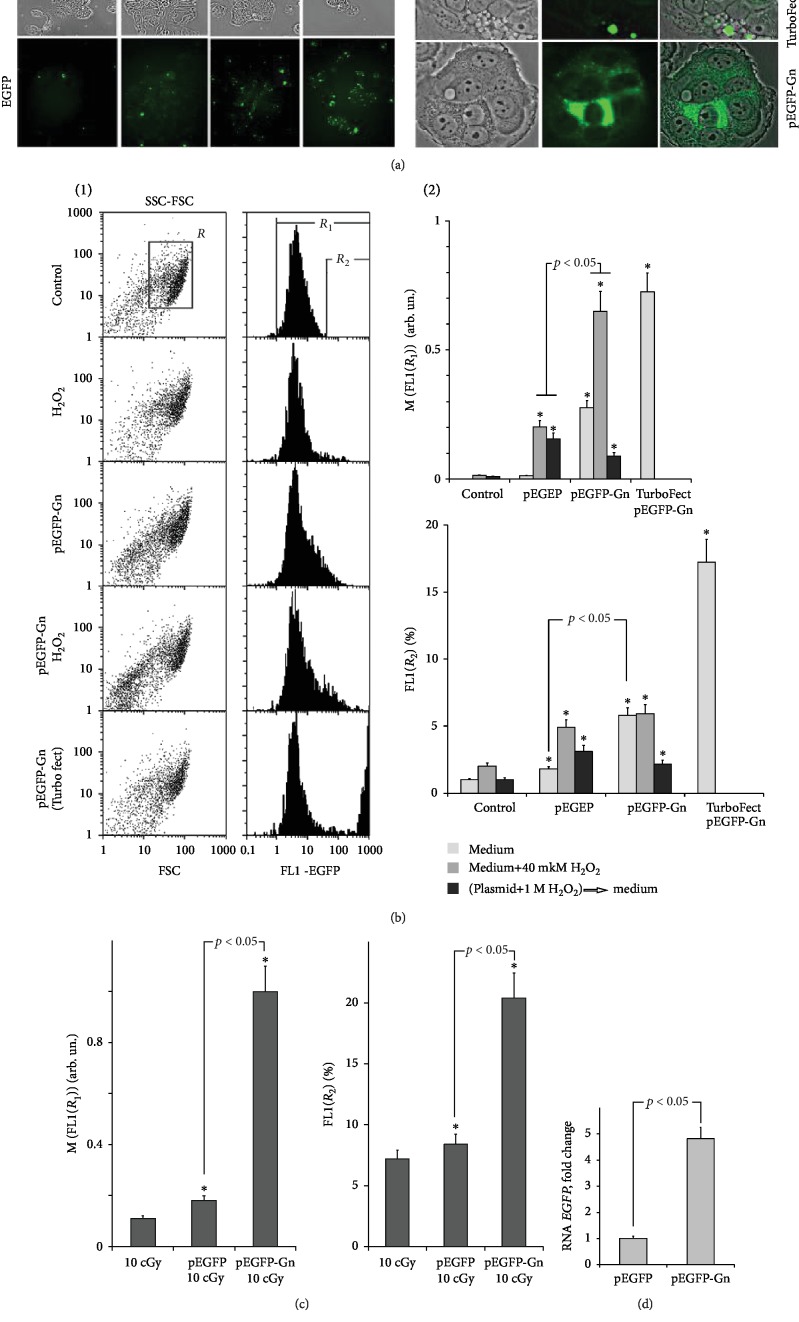
The expression of EGFP in MCF7 treated with pEGFP-Gn or pEGFP for 24 h. (a) FM-based evaluation of EGFP in the cells treated with pEGFP, pEGFP-Gn, or pEGFP-Gn/TurboFect. *λ*_ex_ = 488 nm; magnification ×20 (left) and 200 (right). (b) FCA. (1, left): nonfixed cell plots: SSC versus FCS; *R*: the analyzed cell subpopulation. (1, right): the distribution of FL1-EGFP fluorescence intensities. *R*_1_ and *R*_2_: gated areas. (2) Median signal intensity of FL1-EGFP (*R*_1_) and relative proportions of EGFP-positive cells in *R*_2_ gate; the conditions for cultivation MCF7 and processing of the plasmids are indicated. (c) FCA. Median signal intensity of FL1-EGFP (*R*_1_) and relative proportions of EGFP-positive cells in *R*_2_ gate. MCF7 were irradiated with IR at a dose of 10 cGy. (d) RT-qPCR. The levels of EGFP—encoding RNAs in the cells exposed to pEGFP or pEGFP-Gn. ^∗^*p* < 0.05.

After 24 hours of incubation with pEGFP-Gn, the *EGFP* mRNA level was five times higher than in the case of pEGFP (Figure 7(d)). Protein EGFP in the cells was analyzed by FM (Figure 7(a)). For positive control, cells were transfected with pEGFP-Gn using standard reagent for transfection TurboFect. Population of the control cells contains single cells with autofluorescence. In the presence of pEGFP-Gn or pEGFP, the number of fluorescent cells increases, especially in the case of pEGFP-Gn. At higher magnification, additional differences can be observed (Figure 7(a) (right)). The use of pEGFP-Gn/TurboFect leads to “all-or-none” staining of the cells. There are highly fluorescent cells and virtually unstained cells present. In the case of pEGFP-Gn, the number of highly fluorescent cells and the intensity of staining are lower than with pEGFP-Gn/TurboFect, but there are more cells that contain small amounts of protein EGFP (low-colored cells).

Figure 7(b) shows FCA data for the unfixed cells. Two parameters were analyzed: the number of cells with relatively high levels of fluorescence (gate *R*_2_, Figure 7(b), 1) and the median signal of the cells (gate *R*_1_, Figure 7(b), 1). FCA data confirm the data of FM. In the presence of pEGFP-Gn/TurboFect, about 15% of the cells contain significant quantities of EGFP. Around 7% of the cells treated with 100 ng/mL pEGFP-Gn fall in the region *R*_2_ (Figure 7(b), 2); however, the signal intensity of these cells is much lower. The population of the cells treated with pEGFP-Gn or pEGFP is enriched with the cells expressing small amounts of the protein. Therefore, the median signal of a single cell in case of pEGFP-Gn is relatively strong (Figure 7(b), 1 and 2). In the case of pEGFP, cells express much lower amounts of EGFP (Figure 7(b), 2).

We analyzed *EGFP* expression under the oxidative stress conditions. ROS synthesis in the cells was induced by addition of 40 *μ*M H_2_O_2_ to the medium simultaneously with pEGFP-Gn or pEGFP for 24 hours (Figure 7(b)) or by the single low dose ionizing irradiation (Figure 7(c)). The amount of the cells in the *R*_2_ fraction did not significantly increase in the presence of pEGFP or pEGFP-Gn/40 *μ*M H_2_O_2_. The average signal of the cells increases (Figure 7(b), 2). Single action with IR (10 cGy) after addition of pEGFP-Gn or pEGFP for 30 min significantly increases the expression of EGFP from pEGFP-Gn (Figure 7(c)). Response to pEGFP is less prominent.

A different approach to increase transfection is oxidation of GC-DNAs prior to the introduction of the constructs into the MCF7 culture medium (Figure 7(b)). The plasmids were oxidized with 1 M H_2_O_2_ (30 min, 4°C) and then added to the medium for 24 hours. The concentration of H_2_O_2_ in culture medium was 40 *μ*M. In this case, we observed approximately the same increase in EGFP expression for pEGFP-Gn and pEGFP. The effect was lower than in the absence of prior oxidation of DNA in solution for both plasmids.

## 4. Discussion

The main purpose of this research was to prove that DNA fragments containing dGn motifs have increased transfection and expression and are able to induce an adaptive response in the cancer cells. This hypothesis is based on two experimental facts: (1) increased uptake of oxidized fragments of extracellular DNA in the cancer cells [10] and (2) the elevated levels of ROS synthesis in cancer cells [9].

The pool of human circulating cfDNA is enriched with GC-rich fragments of the genome [15, 16]. The content of GC-rich ribosomal repeats in the circulating cfDNA increases several times in pathology (rheumatoid arthritis [17] and ischemic heart disease [18]) and chronic irradiation [19]. The composition of GC-cfDNA may include CpG islands of many genes, telomeric repeat, mtDNA [20], and the DNA of viruses and bacteria that are enriched with GC pairs. Despite the same GC composition, GC-DNA of the human genome differs from bacterial DNA. Human GC-DNA contains more dGn motifs that are prone to oxidation (Figure 1). The exception is mtDNA, in which the content of dGn is small and equal to the content of dGn in bacterial DNA (Figure 1).

### 4.1. dGn Motifs Enhance the Efficiency of cfDNA Transfection into the Cells

So, human GC-DNA contains a lot of dGn repeats that are easily oxidized. The main source of circulating cfDNA is dying cells [21]. Oxidative stress is one of the main inducers of cell death. It is possible that a part of GC-cfDNA fragments already contains oxidized bases. Nonoxidized GC-cfDNA fragments can become oxidized when approaching the surface of cancer cells that produce elevated levels of ROS.

**Figure 8:**
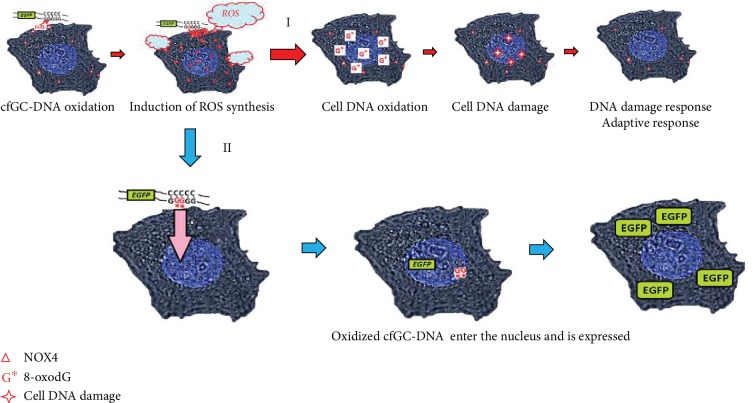
The possible participation of dGn motifs in the implementation of the biological activity of cell-free GC-DNA. A summary of events developing in MCF7 cells exposed to pEGFP-Gn. pEGFP-Gn approaches the cell surface, and dG within dGn is easily oxidized. pEGFP-oxo(Gn) binds to the cell, stimulating *NOX4* expression, synthesis of ROS, and further oxidation of the bases. (I) Cell DNA is damaged by the ROS. The DNA damage response is induced. (II) Oxidized pEGFP-oxo(Gn) is transfected into the nucleus of the cell. Transfection mechanism is still unknown. Perhaps, the yet unknown oxy-DNA sensors are involved in the transfection. *EGFP* gene in pEGFP-oxo(Gn) molecules with intact promoter is expressed.

Genetic construction pEGFP-Gn models the human GC-DNA with dGn motifs.  Figure 8 is a scheme illustrating the interaction of pEGFP-Gn with the cells producing ROS on the cell membrane. When pEGFP-Gn approaches the cell surface, dG in dGn motif becomes selectively oxidized, because it has the lowest oxidation potential compared to other DNA bases [12]. Preferential oxidation of dGn indicates a higher content of 8-oxodG in the composition of the pEGFP-Gn than in the pEGFP, Figure 3(a). Oxidized DNA fragments interact with the cell, stimulating the “burst” of ROS synthesis [10] and further oxidation of pEGFP-Gn and cellular DNA (Figure 4). Functionally important parts of the plasmid containing the promoter and the protein gene *EGFP* are less prone to oxidation as they do not contain long dGn sequences.

One of the enzymes responsible for the synthesis of ROS is NOX4. NOX4 catalyzes the reaction of H_2_O_2_ synthesis. In the presence of pEGFP-Gn, *NOX4* expression is increased (Figure 4). pEGFP-Gn associates with the surface of the cells to form aggregates (Figures 3(d) and 4(d)). These aggregates are surrounded by molecules of NOX4 (Figure 4(d)) and contain 8-oxodG (Figure 3(d)). We have previously obtained evidence indicating the possible stimulation of ROS synthesis upon cfDNA binding with the surface of cancer [10] or stem cells [22].

Oxidized DNA fragments easily penetrate into the cells. This is supported by the fact that pEGFP-Gn is better accumulated in the cells compared to pEGFP (Figure 2(b)) and previously obtained data on the more effective penetration of oxidized DNA into MCF7 cells compared to nonoxidized DNA [10]. The mechanism of penetration of oxidized DNA through the cell membrane into the cell is still unknown. It is possible that some cells express DNA sensors that recognize oxo-cfDNAs. Known DNA sensors such as AIM2 and/or TLR9 can also recognize oxidized DNA. This phenomenon requires further investigation.

### 4.2. dGn Motifs Increase the Expression Levels of the Genes within cfDNA Fragments

For the expression of cfDNA genes to take place, cfDNA fragments must contain an intact promoter of the gene and need to be delivered into the cell. There are several methods of delivering an artificial DNA construction into the cell, including damaging cell membranes and packaging of the DNA constructions into viruses or in lipophilic particles. Oxidative modification of cfDNA prior to the addition of constructions to the culture medium or at the time of their interaction with the cellular surface is another way to enhance the penetration of extracellular DNA into cells and increase the expression of this DNA.

The level of EGFP protein expression from pEGFP-Gn plasmid is higher than from the vector pEGFP and lower than in the case of the standard reagent for transfection (Figures 7(b) and 7(d)). Expression from both plasmids is increased under the action of a weak inducer of ROS synthesis (Figure 7(c)). Apparently, under these conditions, more plasmid molecules become oxidized resulting in more DNA entering the cells. More intense oxidation of the extracellular DNA molecules may be due to oxidation in the culture medium or to increased synthesis of ROS by the cells in response to the action of inducers of ROS.

For efficient expression of the extracellular DNA genes, a balance between the penetration efficiency of DNA in the cell (depends on the oxidation level of the plasmid) and the damage of important DNA regions (in this case promoter and gene EGFP) should be found. An artificial increase in the level of ROS by pretreatment of plasmids with 1 M H_2_O_2_ (Figure 7(b)) apparently causes significant damage to the promotor site of the plasmid. In this case, the expression of the extracellular DNA reduced so that there is no significant difference between the levels of expression of pEGFP-Gn and pEGFP.

### 4.3. dGn Motifs in GC-DNA Induces Adaptive Response in the Cells

In the course of this study, we discovered a very interesting phenomenon. With the appearance of the new dGn-containing DNA fragments in the culture medium of the cancer cells, the number of DNA breaks in the nuclei of the cells sharply increases within a few minutes (Figures 5 and 8). The increase in the number of breaks in nuclear DNA is apparently a consequence of the explosion of ROS synthesis in response to the action of GC-DNAs (Figure 4). It turned out that this response to the action of GC-DNAs is characteristic not only for MCF7 cells but also for human astrocytoma cancer cells (1321N1). Moreover, noncancerous differentiated (HEFs) and undifferentiated (MSCs) cells also increased the number of DNA breaks in response to the presence of the GC-DNA fragments in the medium. The effect of increasing the number of breaks in the DNA of the cancer cells is short term: after 15 minutes, the number of breaks decreases. However, cancer cells long enough remember this effect. Apparently, in this case, we are dealing with the development of an adaptive response. An adaptive response increases the resistance of cancer cells to the subsequent action of the damaging agent. In our case, this is ionizing radiation (Figure 6). This phenomenon requires further investigation.

### 4.4. The cfDNA Role in the Functioning of Cancer Cells

oxo-cfDNA has a pronounced biological activity against the cancer cells MCF7 [9, 23]. Nuclear DNA damage is observed, cell cycle is arrested, and DDR takes place. This reduces the level of cell death and enhances survival in MCF7 cancer cells. In this study, we found that prone to oxidation extracellular DNA not only penetrates into the MCF7 cells and enhances survival of the cells but also can be expressed.

The sequences expressed depend on the cfDNA and its oxidation level. We have previously assumed that in chemotherapy or irradiation-treated human body, the release of oxidized DNA from dying cancer cells may give a boost to remaining malignant cells by augmenting their survival and stress resistance [9]. Apparently, protein and/or RNA expression from cfDNA contributes to the previously described mechanism. In the course of any therapy aimed at destroying cancer cells, the dead tumor cells are a source of new cfDNA molecules. Because of tumor oxo-cfDNA penetration into the cells, new metastases may develop.

The property of the genetic constructions containing easily oxidized motifs to penetrate into the cancer cells and become expressed can be used in complex antitumor therapy. For example, constructions similar to pEGFP-Gn containing genes of proteins or genes of siRNA that blocks proliferation and metabolism of cancer cells can be used during radiation therapy.

## Data Availability

The data used to support the findings of this study are available from the corresponding author upon request.

## Abbreviations

cfDNA: Cell-free DNA
GC-DNAs: A generic term for all DNA probes used in this study
PE: Phycoerythrin
FCA: Flow cytometry analysis
FM: Fluorescent microscopy
mtDNA: Mitochondrial DNA
DDR: DNA damage response.
